# Altered sleep behavior strengthens face validity in the ArcAβ mouse model for Alzheimer’s disease

**DOI:** 10.1038/s41598-024-51560-3

**Published:** 2024-01-10

**Authors:** Alp Altunkaya, Cassandra Deichsel, Matthias Kreuzer, Duy-Minh Nguyen, Ann-Marie Wintergerst, Gerhard Rammes, Gerhard Schneider, Thomas Fenzl

**Affiliations:** Department of Anesthesiology and Intensive Care, School of Medicine and Health, Klinikum Rechts der Isar, Technical University of Munich, Munich, Germany

**Keywords:** Neuroscience, Circadian rhythms and sleep, Diseases of the nervous system, Neural ageing

## Abstract

Demographic changes will expand the number of senior citizens suffering from Alzheimer's disease (AD). Key aspects of AD pathology are sleep impairments, associated with onset and progression of AD. AD mouse models may provide insights into mechanisms of AD-related sleep impairments. Such models may also help to establish new biomarkers predicting AD onset and monitoring AD progression. The present study aimed to establish sleep-related face validity of a widely used mouse model of AD (ArcAβ model) by comprehensively characterizing its baseline sleep/wake behavior. Chronic EEG recordings were performed continuously on four consecutive days in freely behaving mice. Spectral and temporal sleep/wake parameters were assessed and analyzed. EEG recordings showed decreased non-rapid eye movement sleep (NREMS) and increased wakefulness in transgenic mice (TG). Vigilance state transitions were different in TG mice when compared to wildtype littermates (WT). During NREMS, TG mice had lower power between 1 and 5 Hz and increased power between 5 and 30 Hz. Sleep spindle amplitudes in TG mice were lower. Our study strongly provides sleep-linked face validity for the ArcAβ model. These findings extend the potential of the mouse model to investigate mechanisms of AD-related sleep impairments and the impact of sleep impairments on the development of AD.

## Introduction

Alzheimer's disease (AD) has become the 7th leading cause of death for all age groups in the world and, in particular, the fifth leading cause of death for people over 65 years of age^1^. It is expected that 152 million patients worldwide will suffer from AD in 2050^2^.

The key pathological hallmarks of AD are the accumulation of intracellular neurofibrillary tangles made up of hyperphosphorylated tau protein, the extensive deposition of extracellular amyloid plaques across the brain parenchyma, and neuronal loss^3–5^. The preclinical stage of AD, which lasts 10–20 years before cognitive symptoms manifest, is physiologically characterized by the accumulation of Aβ proteins due to an imbalance between its synthesis and clearance, a crucial early step in the pathogenesis of AD^6^. Apart from a small proportion of AD cases caused by autosomal dominant genes (familial AD), AD is considered a multifactorial disease^7,8^.

In recent years, alterations in sleep–wake behavior were proposed to be not only a behavioral symptom of AD pathology but also a facilitator of disease progression, indicating a reciprocal relationship between sleep and early AD biomarkers such as amyloid-beta (Aβ) deposition, tau pathology, circadian Aβ regulation, and glymphatic clearance of Aβ^9^.

Patients with AD suffer from frequent sleep and circadian rhythm disturbances. The intensity of both correlates with disease severity^10^. Patients with no cognitive impairments but with evidence of amyloid plaque formation had a worse quality of sleep than patients without amyloid plaque evidence^11^.

In humans, an experimental loss of sleep directly leads to a modification of cortical excitability^12^. This provides a theoretical framework for a positive feedback loop where impaired sleep triggers cortical excitability, which may lead to increased Aβ accumulation. This would lead to more impaired sleep and a more severe endophenotype of AD. Prodromal AD patients, specifically patients with mild cognitive impairment (MCI), showed longer times in bed, reduced sleep efficiency^13^, and increased rapid eye movement sleep (REMS) latency^13^.

Although the onset and time course of sleep changes from preclinical AD to the onset of mild cognitive impairment in human patients is yet to be defined, animal studies revealed sleep alterations already at prodromal AD-stages. Six-month old Tg2576 mice (amyloid mouse model) showed increased wakefulness and decreased sleep^14^ prior to amyloid plaque deposition^15^ and microglial activation^16^. Further, five-week-old Tg2576 mice showed early hyperexcitability in REMS marked by interictal spikes^17^, a finding paralleled by another study that observed hypersynchronous network activity^18^, underlining the early sleep disturbances linked to β-amyloid neuropathology in this model^17^. Similarly, in the APP/Presenilin 1 mouse model (amyloid mouse model), initiation of amyloid plaque deposition in hippocampus and cortex concurred with increased wakefulness and decreased sleep^19^. Young and aged APPswe/PS1dE9 (APP) mice display perturbations in slow-wave oscillations prior to amyloid deposition^20^.

AD models such as Tg2576 and APP/Presenilin 1 have been pivotal in revealing early sleep alterations in AD and the role of Aβ accumulation, although available data from these models do not fully describe the complexity of sleep/wake behavior in the pathogenesis of AD. The present study was designed to establish a comprehensive insight into temporal and spectral sleep parameter in the ArcAß model (amyloid mouse model), thus providing strong face validity together with the establishment of new EEG-based biomarkers during prodromal stages.

## Material and methods

### Animal model

The ArcAβ mouse model overexpresses the human β-amyloid precursor protein (APP) 695^21^. The mouse model carries, besides the Swedish mutation (APPswe), the Aß associated Arctic mutation (Aß_Arc_), characterized by an exchange of an AA at position AßE22G. The Aß_Arc_ mutation aggravates Aß toxicity by promoting APP processing, Aβ aggregation through facilitating oligomerization. The mice show early signs of AD pathology from 3 months onwards, marked by intracellular Aβ accumulation that peaks between 7 to 9 months. By six months, amyloid pathology affects both the brain parenchyma and vasculature, particularly in the cortex and hippocampus. β-amyloid plaques become noticeable between 9 to 15 months, covering a significant portion of the cortical surface by 21 months^21^. Care of laboratory animals and experiments were performed in accordance with the recommendations of the European Union for the care and use of laboratory animals and ARRIVE guidelines (see below). All experimental procedures were authorized by the Bavarian Government (ROB-55.2-2532.Vet_02-19-120).

### Animal housing

We used fourteen male mice (age: 8–11 month) for the present study (5 ArcAβ transgenic mice (TG), 9 ArcAβ wildtype littermates (WT), Charles River Laboratories, Calco, Italy). No animal showed signs of distress as assessed by the Mouse Grimace Scale: limping, sudden weight loss, lack of fur caretaking, abnormal sleeping positions, bleeding, pus build-up at wound sites, or epileptic attacks. Hence no animal was excluded from the study. Each mouse was kept in individual recording cage with open-top and clear Lucite walls (26 × 26 × 35 cm, custom-made, M. Streicher, Innsbruck, Austria) with the floor covered with wood shaving bedding (Abedd Eypen Classic, ssniff Spezialdiäten GmbH, Soest, Germany) and paper towels for nesting^22^. Food (Altromin 1324 Sp. Beh., Altromin Spezialfutter GmbH & Co. KG, Lage, Germany) and water were provided ad libitum. We performed all experiments within a sound attenuated recording chamber (custom-made, M. Streicher, Innsbruck, Austria) surrounded by a Faraday Cage (type II cage, AMETEK GmbH/TMC, Meerburg, Germany). Animals had olfactory, visual, and acoustic contact. The light–dark cycle was 12–12 h, with Zeitgeber hour 0 (ZT0) corresponding to lights on (200 lx). We kept the temperature at 22 ± 2 °C, and the humidity at 55 ± 10%. Between ZT23-ZT24, daily maintenance of the mouse cage, including food and water replenishment took place, with no change in light intensity.

### EEG/EMG electrode assembly

**Figure 1:**
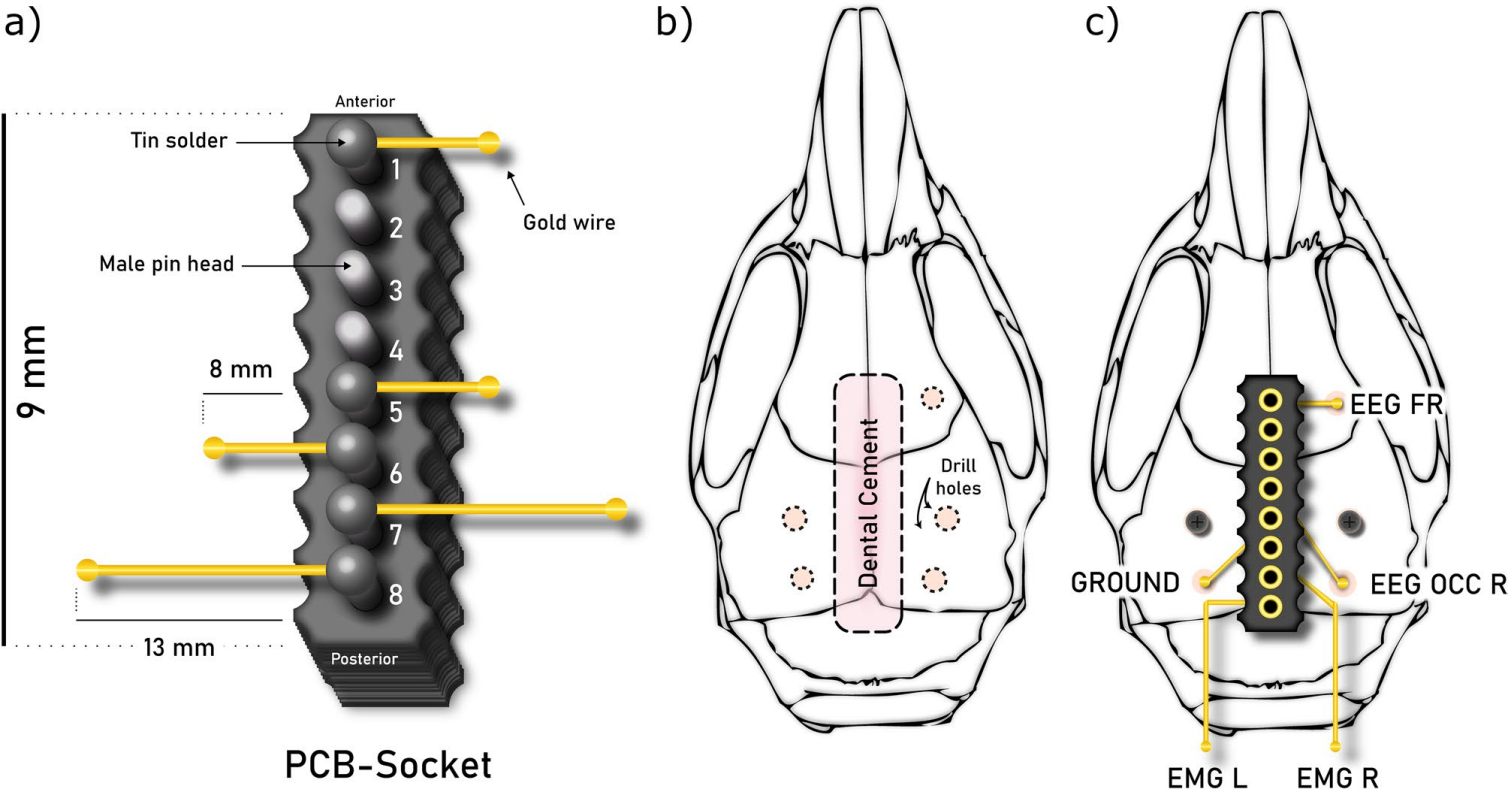
Design of the electrode/headstage connector. (**a**) Printed circuit board (PCB) socket with soldered EEG, EMG and ground electrodes. (**b**) Location of the electrode socket as well as skull penetration locations on the mouse skull. All skull penetrations were placed with reference to bregma and lambda. (**c**) PCB socket with individual electrodes (EEG FR: right frontal EEG; EEG OCC R: right occipital EEG; EMG L & EMG R; left and right EMG) and two screws, mounted on the skull (Coordinates: EEG FR: mediolateral (ML): 1 mm, anterior–posterior (AP): 2.8 mm, dorsoventral (DV): − 0.5 mm; EEG OCC R: ML: 2.8 mm, AP: − 3.5 mm, DV: − 0.5 mm; Ground: ML: − 2.8 mm, AP: − 3.5 mm, DV: − 0.5 mm). The two EMG electrodes were implanted bilaterally (illustration proportions not to scale, mouse skull adapted from^23^).

Electrode-sockets containing electroencephalographic electrodes (EEG) and electromyographic electrodes (EMG) were assembled from gold wire (751 GG gold wire, ø 150 μm; C.Hafner, Wimsheim, Germany), soldered onto PCB-sockets (PRECI-DIP SA Series 861, Delémont, Switzerland; see Fig. 1 for details). Each socket held 2 EEG electrodes 2 EMG electrodes, and a ground electrode. A dental cement (Paladur, Heraeus-Kulzer, Hanau, Germany) cover on the soldering side of the PCB-socket provided extra structural rigidity and electric isolation.

### Surgery for electrode implantation

General anesthesia (GA) was induced in a small plexiglass box (AgnTho's AB, Lidingö, Sweden) with 3 Vol% isoflurane at a flow rate of 190 ml room air/min before the anesthetized animal was transferred to a stereotact (Leica Microsystems, Witzlar, Germany). The nasal chamber was adapted to the anatomy of the mouse (AgnTho's AB, Lidingö, Sweden). GA was maintained with 1.4–2.0 Vol% isoflurane with a flow rate of approx. 190 ml room air/min (Univentor 400 Anesthesia Unit, Angatho´s, Lidingö, Sweden). The animal was placed on a heating pad with feedback control at 37 °C (Homeothermic Monitoring System, Harvard Apparatus, Massachusetts, United States). 125 µl Lidocaine (Lidocainhydrochlorid 2% (diluted to 1 mg/ml), bela-pharm GmbH & Co. KG, Vechta) were applied subcutaneously to add local anesthesia to the surgery site. After shaving the surgical site (cosmetic shaver, Rossmann, Germany) and covering the eyes with eye cream (Bepanthen-Dexpanthenol, Bayer Viral GmbH, Leverkusen, Germany), the scalp was opened. Wound edges were fixed with retractors, and the periosteum was locally removed. Skull penetrations (Ø 500 µm, medical dental drill, type 4811, KaVo, Biberach, Germany) were then placed for each individual electrode and two jeweler’s screws (Ø 0.6 × 2 mm; Paul Korth GmbH, Lüdenscheid, Germany, see Fig. 1). All EEG electrodes were inserted into their respective skull penetrations and placed onto the *dura mater.* EMG electrodes were placed into the *musculus semispinalis capitis* bilaterally through microscopic incisions. Finally, the skull penetrations were sealed with dental cement and the wound was closed with single button sutures. The animals came back into their home-cages and we observed each animal individually until complete recovery after GA, but at least for 15 min. A part of the home-cage floor was heated with a heating pad (custom made) preventing cooling. For postoperative analgesia, the animals received carprofen (0.067 mg/kg, Carprox, Virbac) in the drinking water starting one day prior to surgery and continuing for 4 days post-surgery, with water refreshed daily and consumption of water intake was monitored (approx. 6–8 ml/day/mouse). Thereafter, water, bedding material, chewing sticks, and paper towel pieces for nest building were replenished every three days and weekly, respectively. A sufficiently high signal-to-noise ratio of the EEG/EMG signal was obtained 10 days after the surgery. During the experiment the health status of the animals and food/water consumption was monitored daily.

### EEG/EMG recordings

The electrode socket was connected to a commutator (Precisor Messtechnik München) with a recording cable (custom made, Max Planck Institute for Psychiatry, Munich, Germany), which was attached to a rotatable and weight-balanced swivel system, weight (custom-made, M. Streicher, Innsbruck, Austria). The raw EEG signal was amplified (1000 × amplification, analog band-pass filter: 0.1–128 Hz, custom made, Max Planck Institute for Psychiatry, Munich, German, Munich, Germany) and digitalized (sample rate: 256 Hz, NI-DAQmx, National Instruments, Austin, Texas), All signals were recorded continuously with EGERA recording software (SEA, Köln, Germany). Upon full recovery from the implantation surgery after 10 days, EEG recordings were initiated at ZT0 and continued for 96 h. For data analysis, the 23 h period preceding the final hour of the last 23 h cycle was selected; the last hour was omitted to facilitate daily maintenance of the mouse cage, including food and water replenishment. Mice were recorded in two batches, one comprising 8 and the other 6 male mice, with recordings conducted simultaneously within batches.

### Histological verification

Following EEG/EMG recordings, mice were perfused with ice cold physiological NaCl for 4 min, followed by ice cold formaldehyde solution (4% PFA in 1 × PBS for 8 min). After perfusion, brains were extracted and sectioned into 50 µm thick slices with a microtome. A few sample sections were taken to ascertain the presence or absence of plaques, serving as an additional verification to ensure that the genotyped mice indeed manifested their expected amyloid pathology. Brain slices were then fixed with a 4 °C Aceton-Iso-Propanol solution (1:1) for 20–30 min and washed twice with a 1x-PBS-EtOH and MEK (Methylethylketon) 1:1 solution for 10 min. The slices were stained with Methoxy-X04 in PBS-EtOH solution for 30 min, washed three times with 1x-PBS-EtOH and MEK 1:1 solution for 5–10 min and with ddH2O for 10 min. Finally, the slides were covered (Dako Vector mounting medium H-1400, Vector Laboratories, Newark, CA, United States) and dried for 20–60 min.

### Data analysis: EEG

All EEG/EMG recordings were analyzed using custom-made MATLAB and LabView scripts^24^. Raw EEG/EMG data with .tdms extension were converted to .txt data using the TDMS Reader toolbox (version 2.6, Jim Hokanson, MathWorks File Exchange) and divided into two halves to represent light periods (ZT0-12) and dark periods (ZT12-23). All data were down-sampled from 256 to 125 Hz. To assign EEG signals to individual vigilance states, the following frequency bands were classified: δ (0.5–5 Hz), θ (6–9 Hz), α (10–15 Hz), η (16–22.75 Hz) and β (23–31.75 Hz)^22^. Sleep scored data^24^ were analyzed for differences in basal sleep/wake behavior, spectral and temporal features of EEG data. Sleep/wake behavior was scored by two blinded experts, unaware of the mouse genotypes, using a semi-automated scoring software^24^. Each 4 s epoch of vigilance state classification was extensively reviewed and corrected as needed.

To define the initiation of a bout, a vigilance state transition was recognized only if the signature of the designated vigilance state was sustained for a minimum of three consecutive epochs. No artifacts were observed in our EEG recordings, other than movement-induced artifacts during WAKE. These artifacts were addressed applying a threshold. During this process, an expert sleep scorer delineated a line manually, dictating which data points should be excluded from the subsequent EEG analysis. These movement-induced artifacts had minimal impact on our data, leading to the exclusion of only about 0.015% of the total EEG recordings on average (approximately 13 s per 24 h period).

### Data analysis: spectral features

For calculating power spectral density (PSD) plots, the data were first normalized to ensure each frequency distribution summed to unity. To estimate the variability in the data, bootstrapping with 10,000 iterations was employed. For each iteration, a sample was drawn with replacement from the original data, and the median across frequencies was computed. Following the bootstrapping process, the medians were then used to determine the 95% confidence intervals (CIs) based on the 2.5th and 97.5th percentiles of the bootstrapped medians. Finally, both the median values and their confidence intervals were converted to percentages for visualization.

### Data analysis: temporal features

For calculating vigilance state over time plots, same approach was employed as the PSD plots, in which the median values of each group for every 2 h bins were interpolated to create a line, whereas the CIs were generated using 10k-fold bootstrapping approach.

Graphs illustrating the percentages of vigilance states were generated by calculating the ratio of the duration of each respective bout to the total duration of the predetermined light/dark phase (12 and 11 h, respectively) for each subject mouse.

For the evaluation of bout lengths, the median duration of individual bouts across vigilance states during light and dark phase was computed for each mouse.

Transition probabilities between vigilance states were assessed by enumerating all plausible state conversions, specifically WAKE to NREMS, NREMS to REMS, NREMS to WAKE, REMS to WAKE, and REMS to NREMS. Conversions from WAKE to REMS were deliberately excluded from the analysis, as they do not naturally occur, and their manifestation is indicative of narcolepsy/cataplexy symptoms in murine models.

In the analysis of vigilance state latencies during the light and dark phase, the first five bouts of each vigilance state were evaluated, using the WAKE state as a reference for clarity^25^. We chose to analyze the first five bouts to provide a more comprehensive overview of the mice's vigilance state latencies. The latencies to the onset of successive WAKE bouts were denoted using Δ symbols, leading to designations such as ΔT1, ΔT2, up to ΔT5. Three principal parameters were deduced: the differential latencies (ΔT1–ΔT5), the median bout length of the first five WAKE bouts, and a combined metric, which is the sum of the differential latencies and the median bout length. The median value was then derived for each of these parameters, ensuring a consistent and robust representation of the vigilance state latencies across the subject mice.

### Data analysis: bout length distribution

For each mouse, individual bouts were sorted in ascending order based on their durations, each for the light phase and dark phase. The number of bouts, ranging from the shortest to the longest, was designated as the x-axis, while the corresponding bout durations formed the y-axis. To facilitate comparison across all mice, the number of bouts was normalized by dividing by the total bout count for a given vigilance state, and bout durations were normalized by dividing by the cumulative duration of all bouts for that state. Due to variations in bout numbers among individual mice post-normalization, interpolation was needed to ensure comparability across subjects. Each mouse's data was upsampled to 1000 points for consistent comparison. The median from all mice was taken for each group's representation, while the first and third quartiles were used to depict error bands.

### Data analysis: sleep spindle detection

Analysis was performed using an automated MATLAB-based paradigm for the detection of sleep spindles (SP) from mouse EEG recordings^26^. Detection parameters: minimum SP duration: 0.5 s, maximum SP duration: 2 s, inter-SP interval: 0.1 s. Raw EEG traces were first bandpass filtered between 10 and 15 Hz (Butterworth band-pass filter: first stopband frequency: 3 Hz, first passband frequency: 10 Hz, second passband frequency: 15 Hz, second stopband frequency: 22 Hz, stopband attenuation 24 dB/octave). The root mean square (RMS) of the filtered EEG was calculated using a 750 ms window. RMS values were then cubed to enhance the signal-to-noise ratio on the y-axis and facilitate threshold definition. A two-threshold approach was used to establish inclusion criteria for SP detection during NREMS (lower threshold: 1.0 mean cubed RMS; upper threshold: 2.5 mean cubed RMS). SP were analyzed for duration, normalized amplitude, and SP density (spindle amount divided by NREMS duration [Spindle/min]). Normalized amplitude was calculated by dividing the peak value of the rectified signal envelope to the upper SP detection threshold.

### Statistical analyses

Statistical analyses of all temporal and spectral results were performed using JASP (JASP Team, Version 0.14.1, 2020) and MATLAB (version 2021a, MathWorks, Natick, MA, USA). As assumption of and testing for normality below sample sizes smaller than 40 is strongly discouraged^27^, non-parametric tests were favored. Unless otherwise stated, statistical analyses were performed using Mann–Whitney test for cross-sectional comparisons or Wilcoxon signed rank test for longitudinal comparisons.

For the evaluation of differences in spectral power features, calculation of the area under the curve (AUC) of the receiver-operating characteristic (ROC) for each bin with a frequency resolution [(125/512) Hz] of PSD and 10k-fold bootstrapped 95% CIs were performed using the MATLAB-based MES toolbox^28^. For vigilance state over time, the AUC and 95% CI values for each 2 h bin was computed. AUC was defined as a strong classifier if the AUC > 0.7 or AUC < 0.3, and if the 95% CIs did not include 0.5, which also led to the difference between two distributions being considered as significant^28^. AUC was defined as a good classifier if the AUC > 0.7 or AUC < 0.3 irrespective of 95% CI range^29,30^. The significance level was set to *p* < 0.05. All data are presented as individual data points and/or median and CIs unless stated otherwise. All significant and non-significant statistical data along with AUC effect size calculations are listed in supplementary table 1. To statistically assess differences in bout length distributions between the two groups, the Mann–Whitney U (MWU) test was employed to discern any significant distribution discrepancies. For multiple testing correction, the Benjamini-Hochberg (BH) method was utilized to control the false discovery rate (FDR). *P*-values were adjusted using the BH procedure, with a default FDR threshold set at 0.05. Specifically, the largest rank $k$ was determined where $P(k)\le (k/m)\times q$, with $m$ being the number of tests and $q$ the FDR threshold.

## Results

### Baseline sleep properties

**Figure 2:**
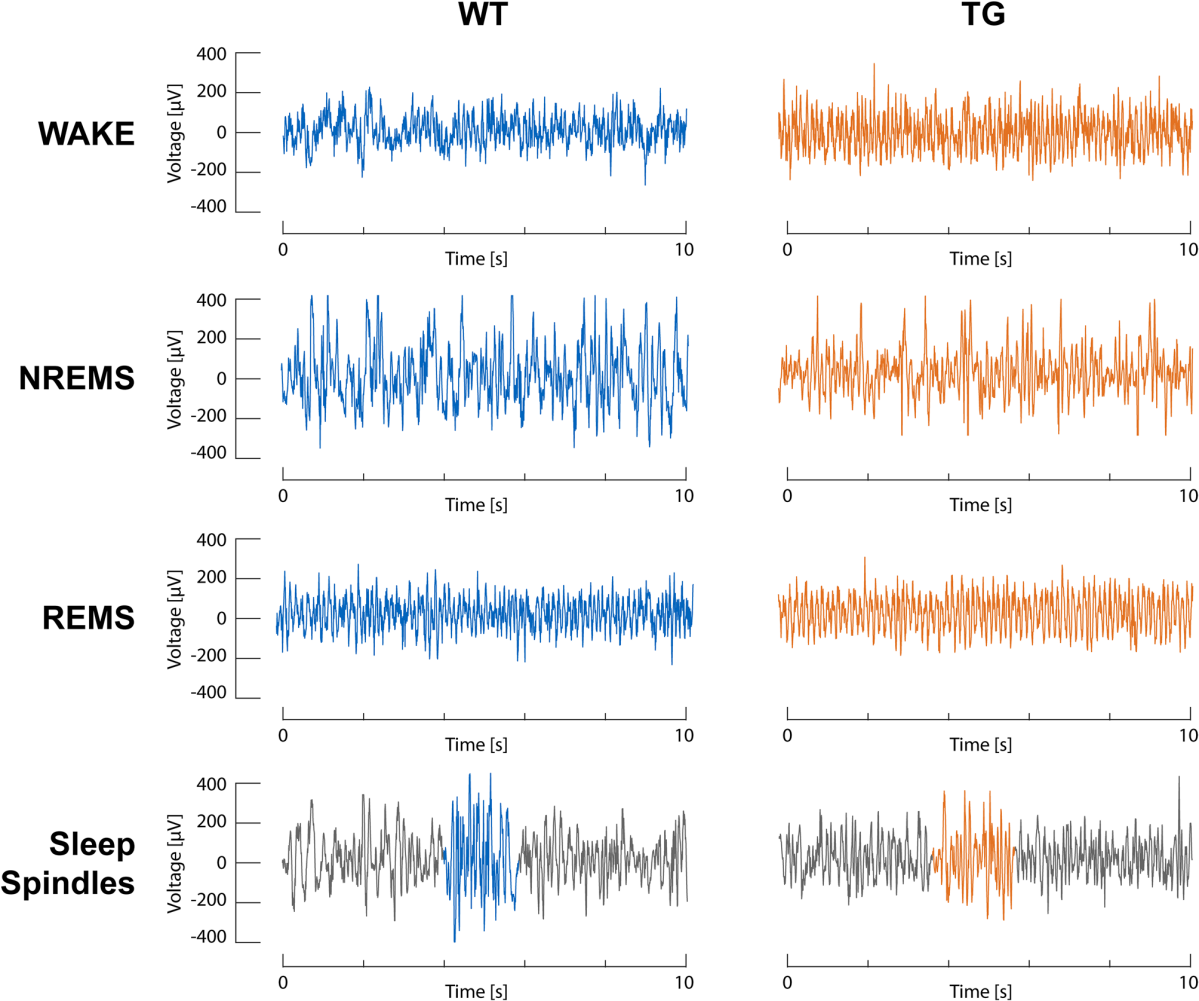
Raw EEG recordings from WT and TG animals. In the ArcAβ model, the general appearance of the EEG is typical for mice. Wakefulness, NREMS and REMS can be separated and scored straight forward. SP are partly visible already before filtering.

All vigilance stages could be separated reliably in the raw EEG from the ArcAß model during scoring (see Fig. 2 for corresponding examples).

**Figure 3:**
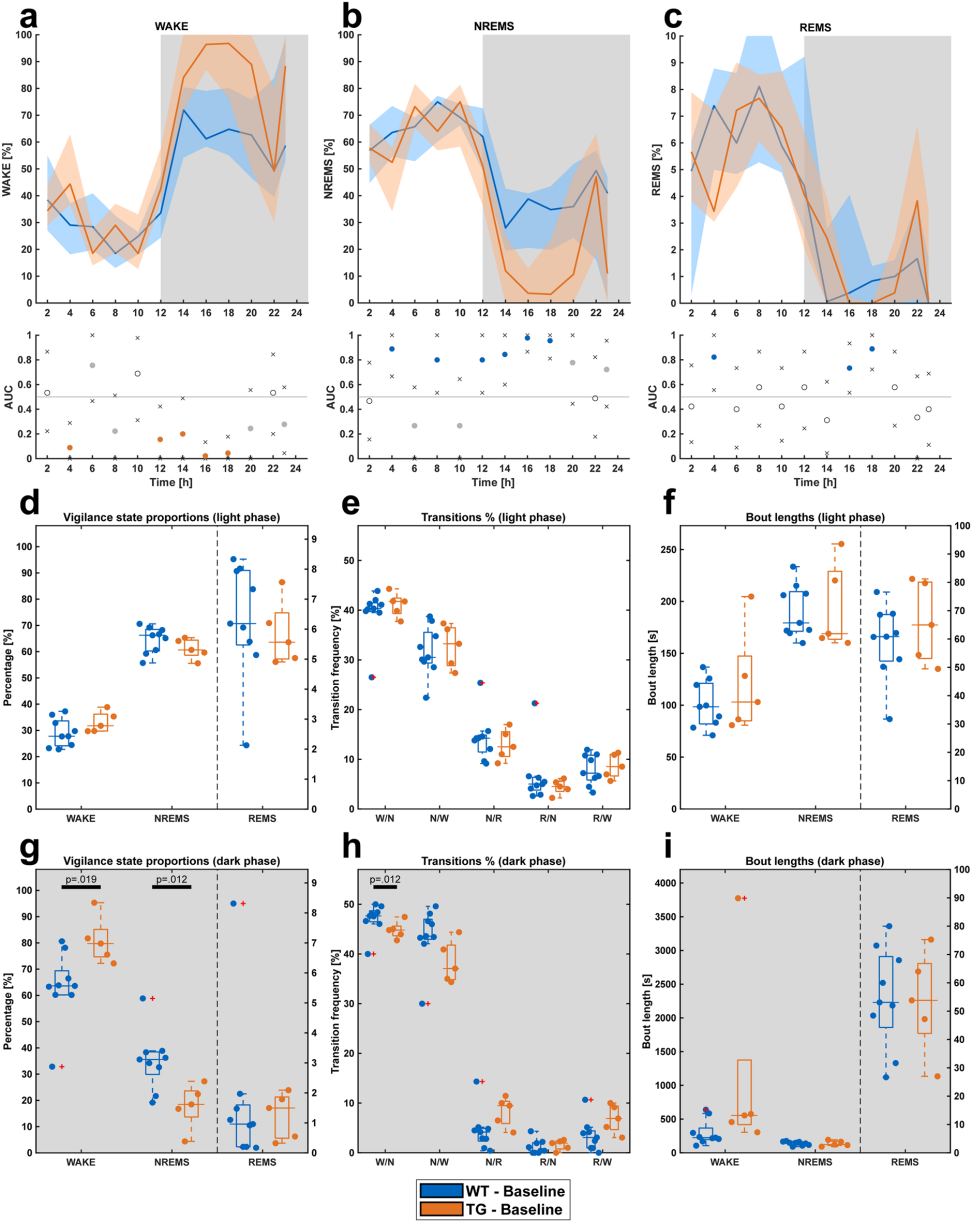
Sleep architecture of WT (n = 9) and TG (n = 5) mice. (**a**–**c**) Overview of vigilance states across the 23 h period. (**d**) Proportions of vigilance states during the light phase. (**e**) Proportions of vigilance state transitions during the light phase. (**f**) Bout lengths of vigilance states during the light phase. (**g**) Proportions of vigilance states during the dark phase, showing a significant increase in WAKE and a decrease in NREMS for TG mice. (**h**) Proportions of vigilance state transitions during the dark phase, revealing altered transition proportions for TG mice. (**i)** Bout lengths of vigilance states during the dark phase, with TG mice displaying longer WAKE bout lengths. For figures a-c: All graphs show median vigilance state percentage values for each individual 2 h bin interpolated into lines, and the shaded areas represent the 10k-fold bootstrapped 95% confidence intervals. Secondary graphs below the 23 h vigilance state graphs show the area under receiver operating characteristic curve (AUROC) values. The “x” symbols represent the lower/upper 95% CIs, whereas gray dots represent when the AUC value is considered as a good classifier (AUC > 0.7 |< 0.3) for the given 2 h bin. The colored dots signify that the confidence interval of a given area under the curve (AUC) value does not include 0.5, which is an indicator for a strong classifier. For all figures showing floating bars and box plots: Middle lines within each box signify the median of the data for showcasing the central tendency. Box edges represent the 1st (25th percentile) and 3rd quartiles (75th percentile), illustrating the interquartile range and data dispersion. Whiskers capture the dataset's minimum (Q1 − 1.5*IQR) and maximum (Q3 + 1.5*IQR) values, delineating the overall range, excluding outliers. Outliers, marked as red plus signs, highlight values significantly deviating from the rest of the data. Gray background indicates the lights-off period (dark phase). Statistical significance was determined with a Mann–Whitney U test for Figures **d**–**i**. For the determination of effect size for all dataset comparisons, AUROC was calculated. Only significant differences are indicated, all other p-values and AUC-values are summarized in supplementary table 1.

After sleep scoring and analysis, the four consecutive days of EEG recording exhibited no significant differences. Consequently, our analyses were confined to a single day 23 h EEG data. Sleep/wake behavior across 24 h was generally distributed as in standard C57BL/6 mice with slightly decreased amounts of wakefulness in the C57BL/6 line during the dark phase, affirming the consistency of sleep/wake patterns in the WT group with established murine sleep behavior norms (data not shown). However, significant differences between genotypes were evident in a multitude of baseline sleep/wake features especially during the dark phase (Fig. 3a–c). No significant differences were observed within the light phase vigilance state proportions (Fig. 3d). Within the dark phase, TG mice showed a significant increase in WAKE (Fig. 3g, *p* = 0.019) with a concomitant significant decrease in non-rapid eye movement sleep (NREMS) proportion (Fig. 3g, *p*  = 0.012).

Vigilance state transitions: Light phase transition proportions did not show differences (Fig. 3e), however, during the dark phase, TG mice showed a significantly decreased WAKE to NREMS transition proportion (Fig. 3h, *p* = 0.042). Further, both NREMS to rapid eye movement sleep (REMS) (Fig. 3h, AUC = 0.200 ± 0.289) and REMS to WAKE (Fig. 3h, AUC = 0.200 ± 0.289) transitions revealed considerable, although non-significant increase in the TG mice compared to its WT littermates.

Bout length and latency: Bout lengths during light phase were not significantly different between WT and TG mice (Fig. 3f). During dark phase, TG mice had a small, non-significant longer WAKE bout length (Fig. 3i, AUC = 0.178 ± 0.266). Distribution of bout lengths between WT and TG mice did not show any discrepancy for all vigilance states, across the 23 h. Latency to any given vigilance state within both halves did not show any significant differences between WT and TG mice (Supplementary material, Fig. 1).

**Figure 4:**
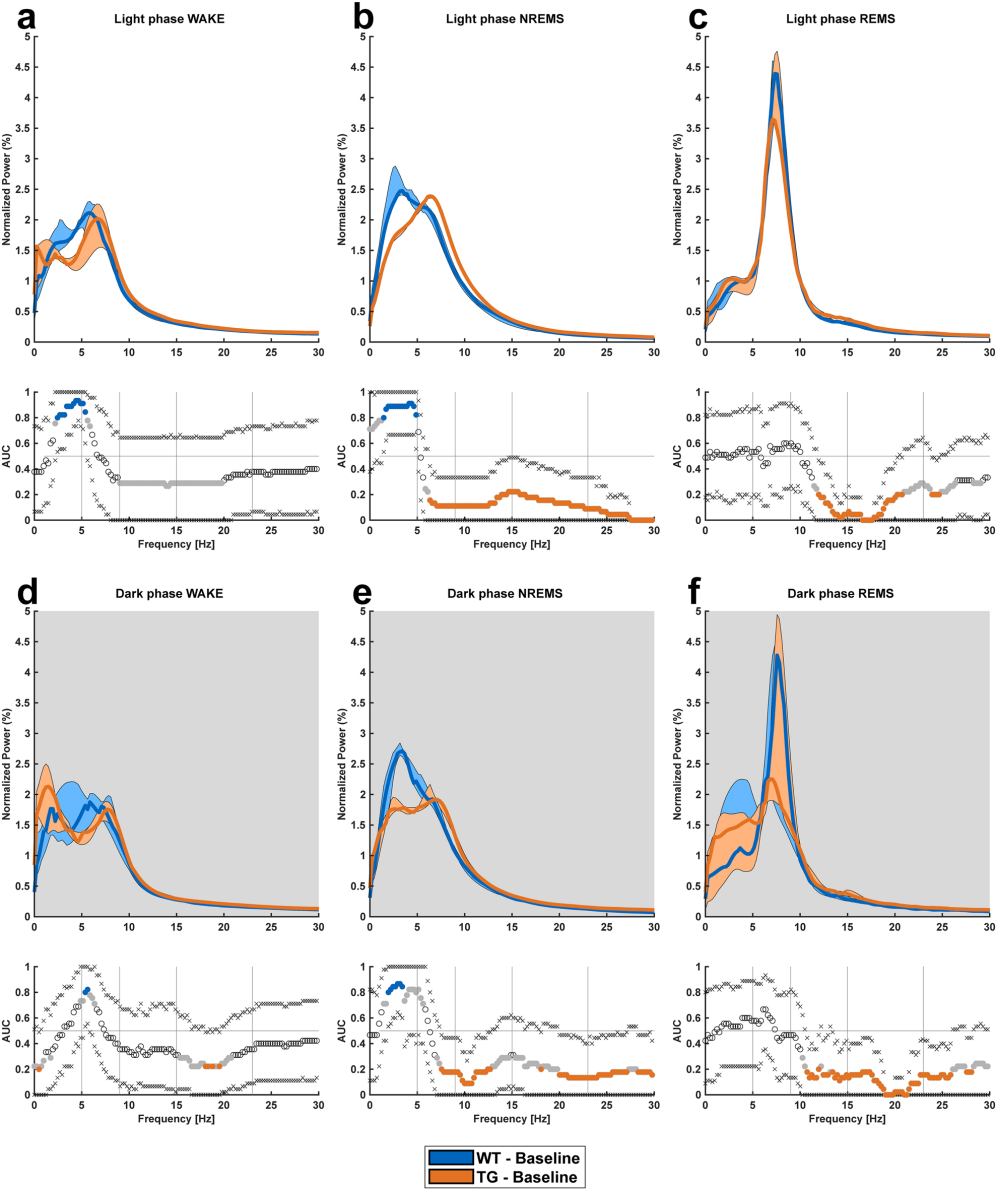
Power spectral density (PSD) in the 1–30 Hz frequency range (frequency resolution. 257 bins with one bin containing a frequency interval of approx. 0.2441 Hz). AUC and 95% CI presented for each vigilance state and phase for WT (n = 9) and TG (n = 5) mice during EEG recordings. (**a**) Light phase WAKE, showing decreased delta power in TG mice. (**b**) Light phase NREMS, with TG mice exhibiting a lower delta power and increased power in theta, alpha, eta, and beta frequency bands. (**c**) Light phase REMS, indicating increased power between 13 and 20 Hz in TG mice. (**d**) Dark phase WAKE, with comparable spectral properties between WT and TG mice. (**e**) Dark phase NREMS, revealing decreased delta power and increased power in remaining frequency bands for TG mice, similar to the light phase. (**f**) Dark phase REMS, showing increased power in alpha, eta, and beta bands for TG mice. For all figures showing PSD values and AUC graphs: All graphs show median PSD values for each individual frequency bin interpolated into lines, and the shaded areas represent the 10k-fold bootstrapped 95% confidence intervals. Secondary graphs below the PSD graphs show the area under receiver operating characteristic curve (AUROC) values. The “x” symbols represent the lower/upper 95% CIs, whereas gray dots represent when the AUC value is considered as a good classifier (AUC > 0.7 |< 0.3) for the given frequency bin. The colored dots signify that the confidence interval of a given area under the curve (AUC) value does not include 0.5, which is an indicator for a strong classifier. Gray background indicates the lights-off period (dark phase).

Genotype-based differences were also present in the spectral properties of the recorded signals. During light phase NREMS, TG mice had a substantially lower power in the delta band, with a noticeable increase of theta, alpha, eta and beta frequency bands compared to WT mice (Fig. 4b). The decreased delta power of TG mice persisted during WAKE state as well (Fig. 4a). Within light phase REMS, TG mice showed an increased power between 13 and 20 Hz (Fig. 4c). Within the dark phase, spectral properties of WAKE remained comparable between WT and TG mice (Fig. 4d). However, similar to light phase, albeit not as prominent, TG mice showed a decreased delta power along with increased power in remaining frequency bands during the dark period NREMS (Fig. 4e). Dark phase REMS showed increased power in alpha, eta and beta bands within TG mice compared to its WT littermates (Fig. 4f).

**Figure 5:**
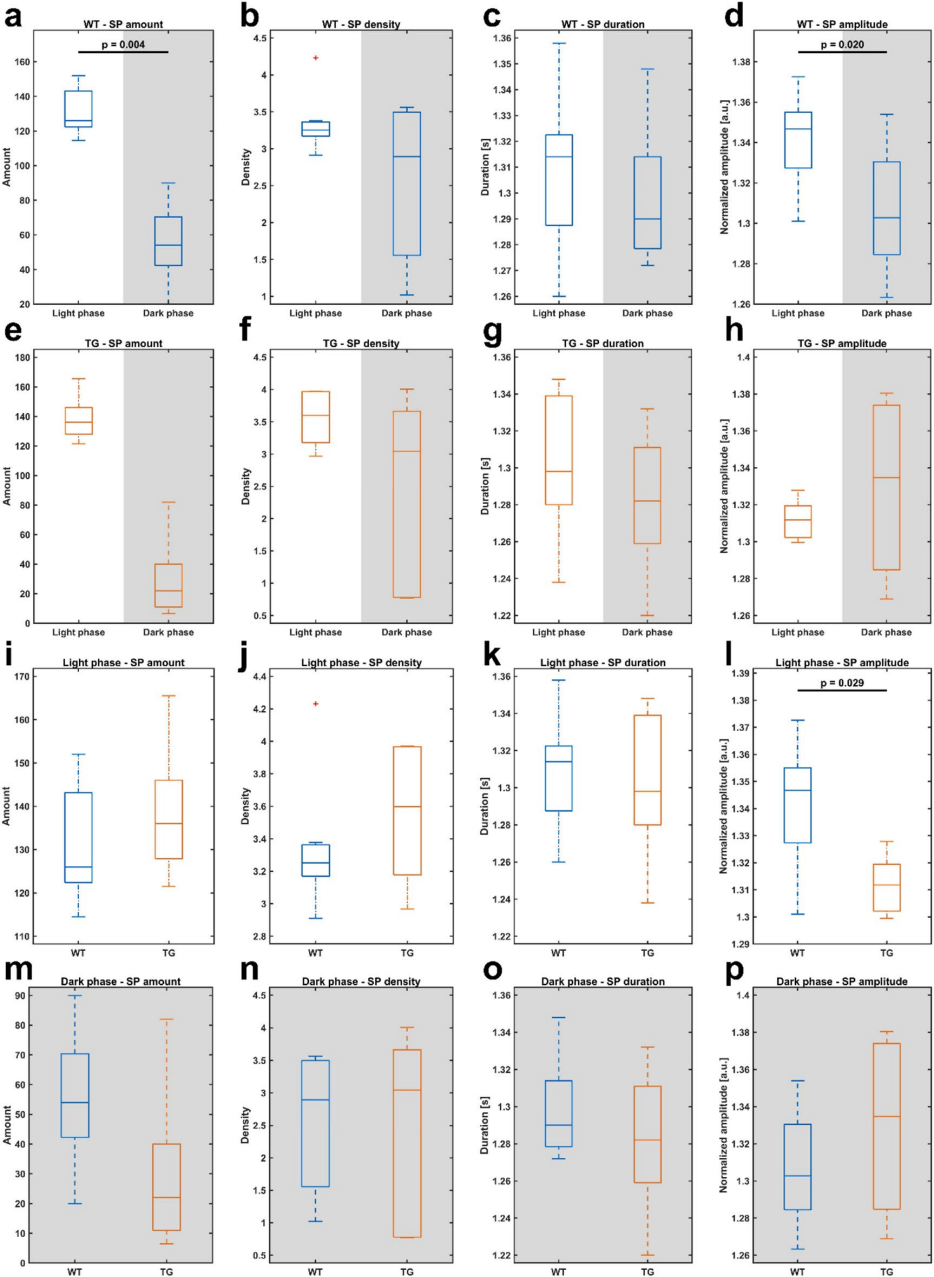
All SP feature comparisons between light and dark phase (**a**–**h**), as well as between WT and TG mice (**i**–**p**). SP amount (**a**, **e**, **i**, **m**), SP density (**b**, **f**, **j**, **n**), SP duration (**c**, **g**, **k**, **o**) and SP amplitude (**d**, **h**, **l**, **p**) were depicted. (**a**) Comparison of SP amount between light and dark phases in WT mice, showing a significant decrease in the dark phase. (**d**) Comparison of SP amplitude between light and dark phases in WT mice, indicating a significant decrease in the dark phase. (**e**) Comparison of SP amount between light and dark phases in TG mice, revealing a decline in the dark phase (*p* = 0.063, AUC = 1.000 ±  < 0.001). (**l**) SP amplitude during the light phase, with a significant decrease in TG mice. Middle lines within each box signify the median of the data for showcasing the central tendency. Box edges represent the 1st (25th percentile) and 3rd quartiles (75th percentile), illustrating the interquartile range and data dispersion. Whiskers capture the dataset's minimum and maximum values, delineating the overall range, excluding outliers. Outliers, marked as red plus signs, highlight values significantly deviating from the rest of the data. Panels **c**, **g**, **k**, and **o** share a common Y-axis range, as do panels **d**, **h**, **l**, and **p**, none starting at 0 for enhanced data representation. Statistical analysis of figures a-h was performed with Wilcoxon signed rank test, Mann–Whitney-U test was used for figures **i**–**p**. Only significant differences are indicated, all other p-values and AUC-values are summarized in supplementary table 1.

Sleep spindles: In TG mice, SP amplitude revealed a significantly decreased amplitude compared to WT mice (Fig. 5l, *p* = 0.029). WT mice showed significantly decreased SP amount in dark phase compared to the light phase (Fig. 5a, *p* = 0.004). Similarly, TG mice showed a noticeable decline in SP-amount in dark phase, compared to light phase (Fig. 5e, AUC = 1.000 ±  < 0.001). WT mice also had a significant decrease in SP-amplitude in the dark phase in comparison to the light phase (Fig. 5d, *p* = 0.020). All other SP features were not significantly different between WT and TG mice, or between light and dark phase within each individual mouse group (Fig. 5).

In the histological analysis, the presence or absence of amyloid plaques in the sample sections corresponded with the expected genotypes.

## Discussion

Neurodegenerative diseases, including AD have long been associated with altered sleep patterns^11^. As recent findings suggested alterations in sleep–wake behavior as a facilitator of disease progression^9^, early sleep impairments may be used in the future as a diagnosis tool. The present study evaluated the face validity of comprehensive sleep characteristics in the ArcAβ mouse model providing a solid tool in fundamental research.

Prominent results in the present study were the significant temporal changes observed in TG mice during the dark (active) phase, TG mice expressed more WAKE and less NREMS. Such findings are well documented in AD patients^31,32^ and animal models^33^. The reduced number of transitions from NREMS to REMS, as found for the TG mice, is different to what has been reported from other AD mouse models such as the 3xTg-AD mouse with an increased sleep fragmentation, leading to increased amyloid-beta levels^33^. Similarly, increased amyloid-beta levels together with increased tau could also be elicited through experimentally induced sleep fragmentations^34–37^. Whether fewer vigilance state transitions contribute to a reduced or retarded amount of amyloid-beta is not known. If so, normalized sleep behavior and consequent sleep hygiene could contribute to slow down disease progression. Such direct impact of sleep behavior on AD pathophysiology was shown for sleep impairments such as insomnia, increased sleep latency, reduced sleep amounts, and disturbed circadian rhythms^38–41^, all increasing the risk for AD.

The comparison of sleep quality between WT and TG mice, as expressed by the slow wave activity (SWA) during slow wave sleep (SWS)^42,43^ revealed a significantly decreased SWA in the TG mice. Clinical studies revealed this loss of sleep quality, showing significant spectral changes during SWA in AD^44^. Interestingly, specific disruption of SWA led to increased amyloid-beta levels in the cerebrospinal fluid^45^. This effect was reported only for SWA disruption and not for total sleep time, NREMS time, REMS time, or sleep efficiency. Disruptions in SWA also seem to be associated with impaired memory consolidation, a process that is fundamental to learning and memory retention^46^, especially also for declarative memories^47^. These associations not only highlight the potential consequences of SWA disruption but also emphasize its influence on cognitive performance. Impaired memory retention even without learning deficits, as observed in ArcAβ mice^21^ could potentially be correlated with alterations in SWA.

TG mice showed a global increase in high frequency power during NREMS and REMS indicating hypervigilance, as such spectral changes were associated with increased arousal during sleep in insomnia patients^48^. Related to this, in AD patients, insomnia and increased arousal affected sleep quality and contributed to sleep disturbances^37^. The increased high-frequency power during sleep, however, is in contrast to findings in AD patients showing a general EEG-slowing and an overall reduction in alpha band power, potentially correlating to increasing Aβ-levels^49^. A similar decrease in the beta band, accompanied by increases in the theta band and delta band were reported^49,50^ along the parthenogenesis of AD^50^. To date, no comprehensive study on temporal and spectral features of sleep impairments along the complete AD-parthenogenesis in the ArcAß is available. An EEG-slowing may be present during late stages in this animal model. Indeed, inconsistent findings have been reported for several AD animal models^51–54^. Especially REMS investigations show variable impairments and its correlation with physiological or behavioral impairments in AD are not as consistent as in human studies^55^.

TG mice had a significant decrease in SP amplitude during the inactive (light) phase, compared to WT mice. A meta study summarizing 22 clinical trials^56^ linked SP amplitudes with general cognitive abilities. Other studies revealed that SP amplitude along with SP density were significantly reduced in AD patients^57,58^. Reduced SP amplitudes were also reported for patients with amnestic mild cognitive impairments^57^. SP have also been positively correlated with enhanced synaptic plasticity in cortical circuits^59^, while cognitive deficits manifested throughout AD progression seem to initially originate from imbalances of cellular and molecular mechanisms of synaptic plasticity^21^. The 3xTg-AD mouse model showed that cognitive impairments correlated with the accumulation of intraneuronal Aβ in the hippocampus and amygdala^60^. Although only a few studies of SP in animal models during AD are available^55^, the presented data support very recent findings^61^ suggesting SP as an additional early EEG-based biomarker for prodromal AD stages and as a general tool in research of dynamic cognitive impairments in AD.

Despite the absence of statistical significance, an elevation in SP amplitude in the TG mice was present during the active phase (Supplemental Fig. 2p). This observation may imply an adaptive response compensating the diminished SP amplitude observed during the inactive phase.

### Conclusions

The present study provides a comprehensive view on relevant temporal and spectral sleep/wake parameter, providing strong face validity to investigate sleep alterations along preclinical AD in the ArcAß mouse model for AD. Sleep impairments of this model partly differ from other established AD models, adding to the toolbox of animal models for AD to investigate all aspects of sleep/AD-pathophysiology interactions.

SP characteristics hold the strong potential to become a valid biomarker for prodromal AD parthenogenesis.

### Limitations

In rodents, sleep architecture and the circadian characteristics differ from humans to a large extent. Differences in sleep behavior may not fully represent the sleep alterations and pathophysiological processes of AD patients. Further, this study utilized an uneven number of mice in both groups, and the disparity in group size, particularly the smaller number in the TG group, might introduce potential biases and contribute to the observed within-group variability in some of our outcome measures. Additionally, the presence of outliers, particularly in vigilance state proportions during the dark phase, could also influence our findings.

More importantly, the relationship between amyloid plaques, tau tangles, and Alzheimer’s disease is not fully elucidated. Although these pathologies are hallmark features of Alzheimer’s, the exact causative pathways remain a topic of ongoing research. The present study did not investigate long-term sleep/wake behavior alterations observed over a period of several months. This could provide a more comprehensive understanding of the progressive nature of sleep disturbances in AD.

### Supplementary Information

Supplementary Legends.

Supplementary Figure 1.

Supplementary Figure 2.

Supplementary Table 1.

## Data Availability

Raw data are available upon request to TF.
