# Supplementary material for: Altered sleep behavior strengthens face validity in the ArcAβ mouse model for Alzheimer’s disease
Source: Sci Rep. 2024 Jan 10;14:951. doi: 10.1038/s41598-024-51560-3 (PMC10781983; doi:10.1038/s41598-024-51560-3)
Supplement: Supplementary file 1 — Supplementary Legends. [file 41598_2024_51560_MOESM1_ESM.docx]

Supplementary Figure 1: Vigilance state latencies between WT and TG mice during baseline measurements. WAKE (a, d), NREMS (b, e) and REMS (c, f) latency comparisons within light and dark phase. None of the above-shown comparisons showed any significant difference.

Supplementary Figure 2: Bout length distributions between WT and TG mice. WAKE (a, d), NREMS (b, e) and REMS (c, f) distribution comparisons within light and dark phase. None of the above-shown comparisons showed any significant difference.

Supplementary Table 1: Statistical analysis results for WT and TG comparison. For all tables showing statistics: vigilance state proportion, bout lengths, transition proportion, between-group SP comparison, within group light vs. dark phase SP comparison, and vigilance state latencies were tested for the null hypothesis that the distribution and median value of the two groups were the same. Columns 1 and 2 show the median value of group 1 followed by its sample size, whereas columns 3 and 4 stand for the median and sample size of group 2. Column 5 indicates the p-value of the corresponding statistical test. For an effect size comparison, area under the receiver-operating characteristic curve (AUROC) was employed. Column 6 shows the resulting AUC value, with lower and upper 95% confidence intervals, shown in columns 7 and 8, respectively. Mann-Whitney U test was employed for all temporal features of sleep/wake behavior other than the within-group SP comparison, where the Wilcoxon signed-rank test was used.
