# Supplementary figures and images for: Altered sleep behavior strengthens face validity in the ArcAβ mouse model for Alzheimer’s disease
Source: Sci Rep. 2024 Jan 10;14:951. doi: 10.1038/s41598-024-51560-3 (PMC10781983; doi:10.1038/s41598-024-51560-3)

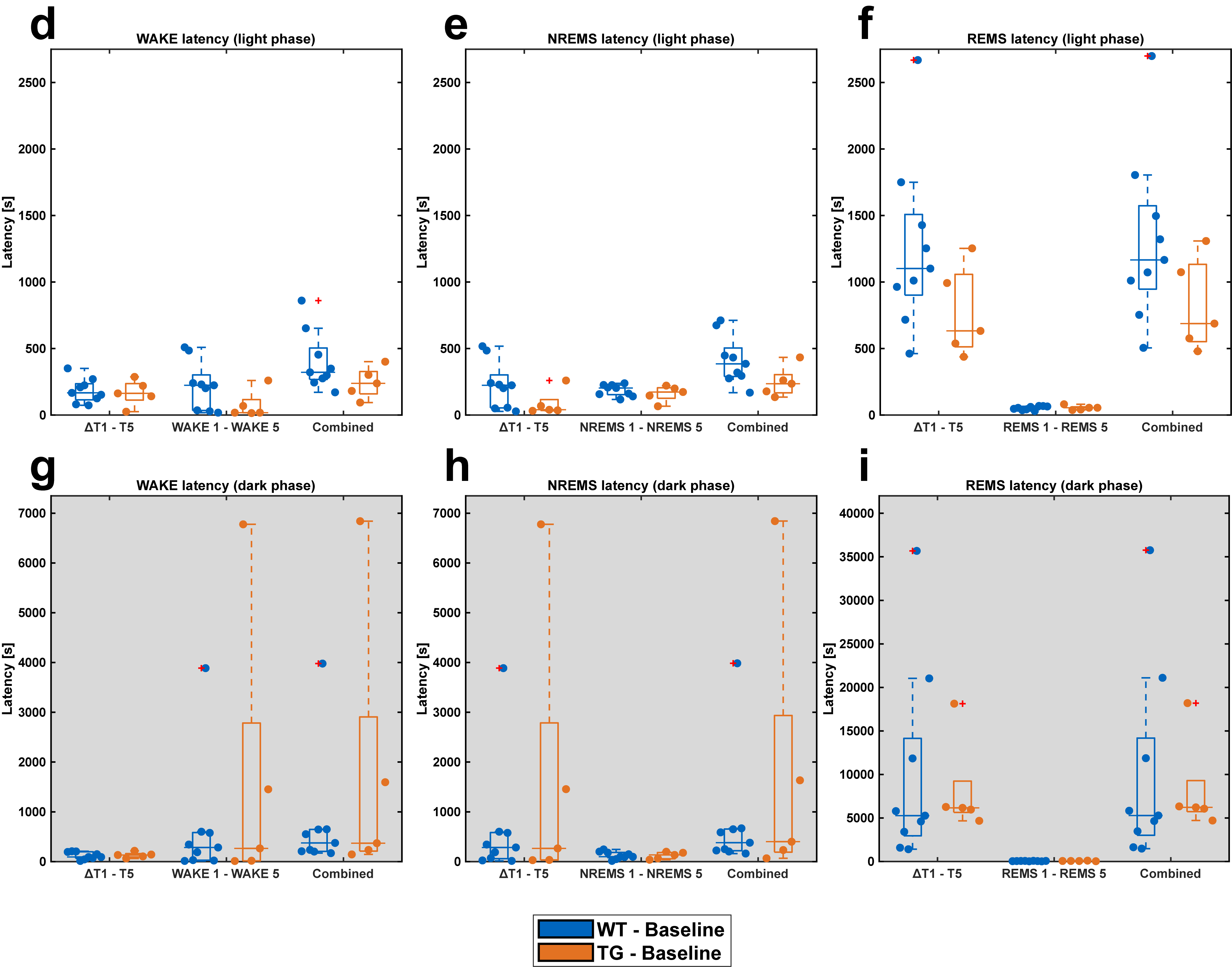

Supplement: Supplementary file 2 — Supplementary Figure 1. [file 41598_2024_51560_MOESM2_ESM.png]

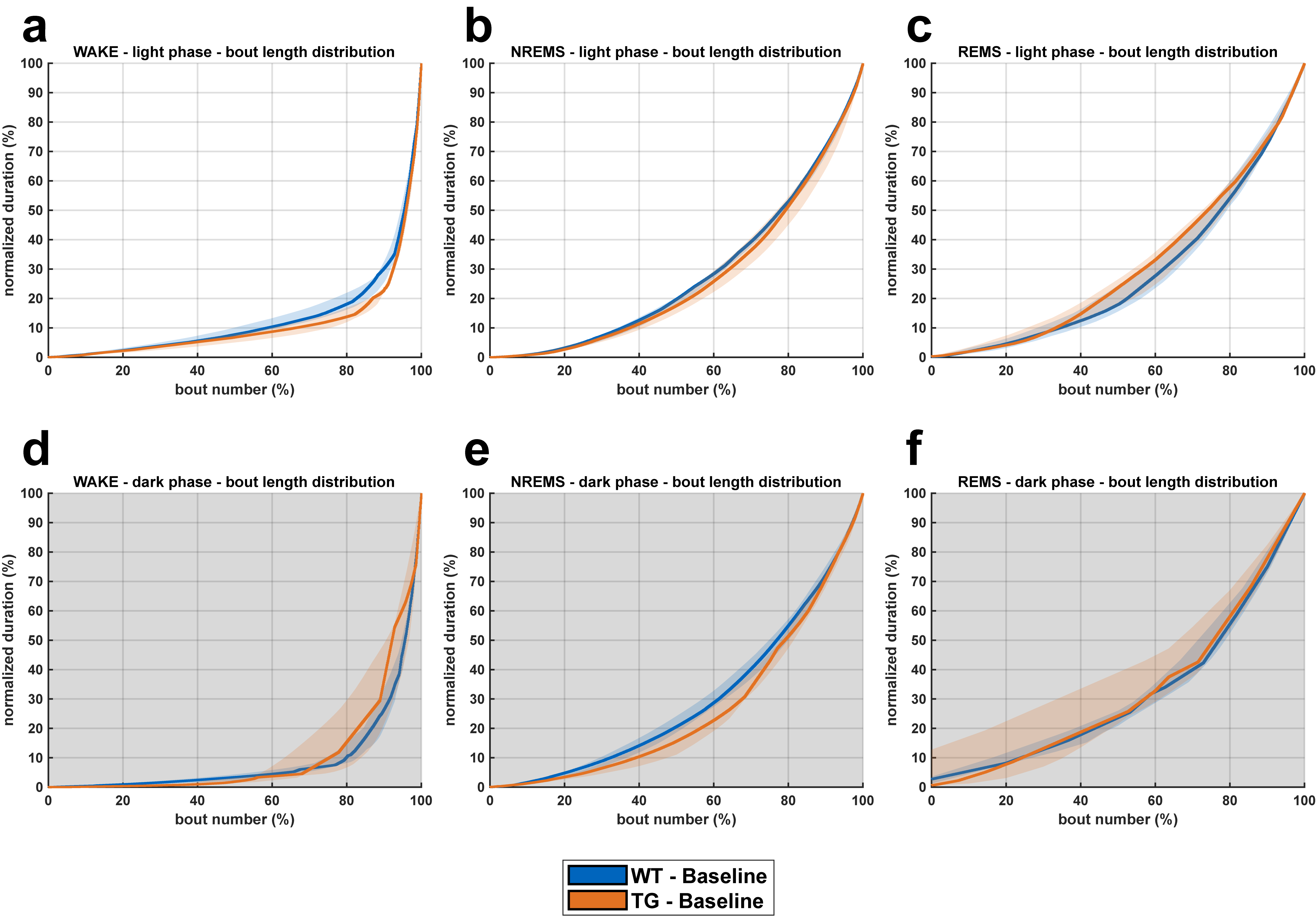

Supplement: Supplementary file 3 — Supplementary Figure 2. [file 41598_2024_51560_MOESM3_ESM.png]
